# Chorasmia Medical School from the beginning until the Mongol invasion

**Published:** 2015-11-14

**Authors:** Seyyed Alireza Golshani, Fatemeh Seddigh, Hadi Pirouzan, Babak Daneshfard

**Affiliations:** 1 PhD Student, Department of History, Faculty of Literature and Humanities- Dr Ali Shariati, Ferdowsi University of Mashhad, Mashhad, Iran, AND Research Office for the History of Persian Medicine, Shiraz University of Medical Sciences, Shiraz, Iran;; 2 Paramedical School, Shiraz University of Medical Sciences, Shiraz, Iran;; 3 PhD Student, Department of History, Faculty of Literature and Humanities, Shiraz University, Shiraz, Iran;; 4 MD, PhD Student in Traditional Persian Medicine, Research Center for Traditional Medicine and History of Medicine, AND Essence of Parsiyan Wisdom Institute, Traditional Medicine and Medicinal Plant Incubator, Shiraz University of Medical Sciences, Shiraz, Iran.

**Keywords:** *Chorasmia*, *historical geography*, *history of medicine*, *medical school*, *Iranian scholars*

## Abstract

In research on the history of medicine, less attention is paid to the subject of historical geography*. *Considering the importance of this subject in the history of science, this paper discusses one of the most important science centers in the world. This outstanding medical research center was located in *Gorganch* city, *Chorasmia* area, in the Eastern part of the Islamic. Chorasmia medical school was one of the important Iranian medical schools before the Mongols’ attack. Its history (305-1231 A.D.) can be divided into three eras; Ale Iraq, Ale Ma'mun, and era of the Khwarazmian dynasty. This geographical area in the Northeast of Iran has escaped the notice of researchers in recent studies. The presence of great Persian physicians and scientists throughout history in this area indicates its scientific importance. The present article focuses on Chorasmia Medical School since its establishment until the Mongols’ attack.

## Introduction

The honorable history of Chorasmia has undoubtedly been shaped by various educational and experimental links which have formed various scientific doctrines and have played a role in the training of different scientists and scholars. Chorasmia Medical School, with the support and encouragement of *Khwarazm Shahs*, raised many physicians, scientists and scholars in the field of mathematics, engineering, astronomy, philosophy, religion sciences, and medicine.

This piece of research is the first general study about Gorganch, Uzbekistan, and Chorasmia Medical School. The great medical school appeared when the Afrighids or *Ale Iraq* took up the reins and grew in power with the governing of Ma'munids or *Ale Ma'mun*. This medical school flourished with the *Khwarazmian* dynasty, but faded with the Mongols’ attack to Iran, and only its reputable name remains in history.

In this study, we aimed to study the role of the Chorasmia Medical School in the development of medicine and reasons for its flourishing. Another goal was to introduce its scholars and their most important work. 

## Method

For this purpose, library resources were reviewed. Moreover, Google Scholar and Noormags databases were searched using Khwarazm, Gorganch, and Chorasmia keywords both in English and Persian. 

*Geographical characteristics*

In many references, *Gorgan* and *Gorganch* have mistakenly been considered to be the same place. Gorgan, called *Esterabad* in the past, is located in the North of Iran about 80 kilometers to the east of the present Gorgan Province (1). 

**Figure 1:**
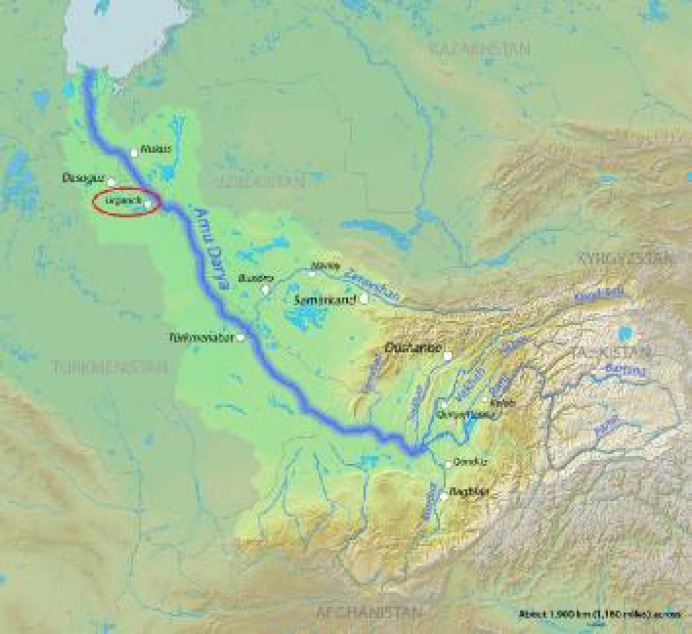
Geographical location of Urgench (Available from: http://en.wikipedia.org/wiki/Amu_Darya)

**Figure 2:**
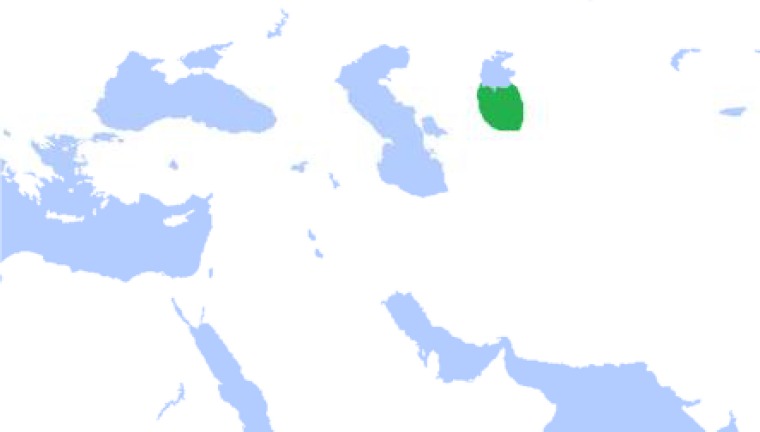
Historical geography of Khwarazm (Available from: http://en.wikipedia.org/wiki/File:AfrighidsMap.png)

*Konye-Urgench* or *Jorjan* or Gorganch, which is called *Jorjanyh* by Arabs, Gorganch by Iranians, and Urgench by the Mongols and Turks, is located on the banks of the *Amu* River (Figure 1). It was considered as one of the most important cities of Chorasmia (2, 3, 4). In the 12^th^ century, Gorganch was the capital of the powerful Khwarazmian dynasty and gained reputation. Since the Khwarazmian dynasty was promoted to the highest level of power in the Islamic world, its capital had to be enriched with treasures of conquered countries (3). *Yaqut Al-Hamawi,* who lived in Gorganch at the end of 1219 A.D. and the beginning of 1220 A.D., believed that Gorganch was the biggest and the richest city ever seen (5). This area even had its own literature and language. According to *Professor Iosef Mikhailovich Oranski*, Khwarezmian (Khwarazmian, Khorezmian, Chorasmian) is an extinct East Iranian language closely related to *Sogdian*^1^*. * The language was spoken in the area of *Khwarezm* (Chorasmia), centered in the lower *Amu Darya* south of the Aral Sea (the northern part of the modern Republic of Uzbekistan, and the neighboring areas of Turkmenistan) (Figure 2) (2,6). 

Our knowledge of Khwarezmian is limited to its Middle Iranian stage and, as with Sogdian, little is known of its ancient form (6). 

From the writings of the great Khwarezmian scholars,* Biruni *and *Zamakhshari,* we know that the language was in use at least until the 13^th^ century, when it was gradually replaced by Iranian in most parts, and several dialects of Turkic (7).

Khwarezm played a vital role in the civilization of central Asia since antiquity, had major cities such as Jorjanyh, Khiva, and Kath, and was seen as the northernmost base for civilization in the past (3, 8). To the present day, Khwarazm and Urgench are still alive as a province and city, respectively, beside the Amu River in the Republic of Uzbekistan (9).

**Historical eras of Chorasmia**

The first example of culture and civilization of Chorasmia is related to the Neolithic Age. It is called “*Kel Teminar*” culture and has been archeologically explored. In the Bronze Age, Khwarazm was the historical site of “*Kokcha-III*”, and in the Iron Age, it witnessed the flourishing of agriculture and industry. Ancient Khwarazm was referred to for the first time in Avesta as “*Iranvij*” which was the birth place of the Aryan tribe (10, 11). This geographical area was under the rule of different empires, from the *Achaemenid *dynasty to Sassanid dynasty, until the advent of Islam (10).

  “The Sogdian language was an Eastern Iranian language spoken in the Central Asian region of Sogdiana, located in modern-day Uzbekistan and Tajikistan. Sogdian is one of the most important Middle Iranian languages, along with Bactrian, Khotanese Saka, Middle Persian and Parthian. It possesses a large literary corpus. The language is usually assigned to a Northeastern group of the Iranian languages, although this is an aerial rather than genetic group. No direct evidence of an earlier version of the language ("Old Sogdian") has been found, although mention of the area in the Old Persian inscriptions means that a separate and recognisable Sogdiana existed at least since the Achaemenid era. Like Khotanese, Sogdian possesses a more conservative grammar and morphology than Middle Persian. The modern Eastern Iranian language Yaghnobi is the descendant of a dialect of Sogdian spoken around the 8th century in Ustrashana, a region to the south of Sogdiana” (6).

The capital of Khwarazm was initially *Kath* *or Cus *(4). Kath was destroyed in the 10^th^ century due to political events between Ale Ma'mun and Ale Iraq and the outflow of the Oxus River. Therefore, the people had to migrate to the east bank of the Oxus River called Gorganch. Chorasmia was an important area and had many cities and towns such as *Xazorasp, Zamakhshar, Drghan, Khiva, Ardkhshish, Safredz, Nozvar, Korder, Kerdran Khvash, Madmineh, Mardjqan, Zaragoza, *and* Otrar* (3, 10). 

The flourishing and development of medicine in Chorasmia, which is known as the original home of the Aryan tribe (10, 11), was actually due to the presence of kings who loved and admired science and governed it for centuries. The advancement of medicine in this part of the world can be divided into three historical eras; the Afrighids or Ale Iraq, Ma'munids or Ale Ma'mun, and the Khwarazmian dynasty.

**First era**

Chorasmia was divided into two parts by the Oxus River (Amu River). Before Islam, from 305 to 892 A.D., both parts were governed by the Afrighids or Ale Iraq who considered themselves as belonging to the *Kai Khosrow Kiani* dynasty (12). Then, in 892 A.D. Khwarazm was broken into two parts. The Afrighids continued to govern the part of Chorasmia which was located in the North of the Oxus River and its capital was *Kath*. However, the South of the Oxus River was governed by a ruler under the title of Amir of Gorganch and the Ale Ma'mun dynasty (13). 

Evidently, the Afrighid dynasty cannot be considered as an independent government, since in 706 A.D., during the Arab conquest, they were entitled to pay tax and continued to do so until the Abbasid Caliphate (14). When *Amr-i Laith Saffari* came into power, the Afrighids lived in harmony with this government at first. However, with *Amr-i Laith’s* defeat, they accepted the rule of *Shah Ismail Samani* and remained obedient until the Ale Iraq dynasty collapsed (14).

According to Al-Biruni, *Afrig* was the first person to be assigned as a ruler in Chorasmia by the Afrighid dynasty. Al-Biruni then talks about the governing of *Bagra*, *Sahhasak*, *Askajamuk* *I*, *Azkajwar I*, *Sahr I*, *Shaush*, *Hamgari*, *Buzgar*, and* Arsamuh *[It is believed that he was contemporary with prophet Muhammad (PBUH)] (12). He subsequently talks of *Sahr II, Sabri*, *Azkajwar II*, *Askajamuk II* (lived during the Islamic conquest), *Sawashfan*, *Torkasbatha*, *Abdollah Ibn Torkasbatha *(the first government of Chorasmia with an Islamic name), *Mansur Ibn Abdollah*, *Eraq Ibn Mansur* (contemporary with Shah Ismail Samani), *Muhammad Ibn Eraq, *and *Abu Sa'id Ahmad Ibn Muhammad* (12). Finally, he talks of *Abu 'Abdollah Muhammad Ibn Ahmad *who was killed in 995 A.D. by *Ma'mun Ibn Muhammad*, the founder of the Ma'munids (12). 

Unfortunately, there is not enough information about Khwarazm Shahs of the Afrighid dynasty. Some researchers even consider this dynasty to be the same as Ale Ma'mun (15). Very little information exists about other scholars and scientists. However, the rulers of Chorasmia were undoubtedly strong supporters of science. Muhammad Ibn Eraq founded the Soltani School of Kath in Chorasmia. Other scientific centers which were known as the “King’s links” were also established. This indicates that the kings of this dynasty supported scientific activities (16). Some of the most famous scientists and physicians of this era were *Al-Gaurizin* (780-850 A.D.) (mathematician, astronomer, philosopher, geographer, and historian), *Al-Biruni* (pharmacologist, historian, mathematician, and astronomer), and *Abu Nasr Iraqi* (mathematician and astronomer). After the collapse of the Afrighid dynasty, many of them continued to live under the rule of Ale Ma'mun (17).

**Second era'**

The second era started with the ruling of the Ale Ma'mun, an anonymous dynasty of Chorasmia’s local rulers. Their name appeared in history in about 995 A.D. and they governed Chorasmia until 1017 A.D. The capital of Ale Ma'mun was Gorganch or Jorjanyh (18).

At first, Ma'munids, or Ale Ma'mun, were under the rule of the Samanids. After the collapse of the Samanid dynasty, they governed independently for a short period of time (18).

By the rise to power of the *Ghaznavids*, they were under the rule of Mahmud of *Ghazni,* and with his attack in 1017 A.D., this golden era came to an end (18). As previously mentioned, the Khwarazm Shahs loved and supported scientific activities, but there is no information about the ancient kings of this dynasty and only the names of four kings are mentioned in history. These rulers were *Abu-Ali Ma'mun I* (ruled in 997), *Abu al-Hasan Ali Ibn Ma'mun* (ruled 997-1008), *Abu Abbas Ma'mun II* (ruled 1008-1017), and *Abu'l-Harith Muhammad* (ruled in 1017) (19). The rulers of Ale Ma'mun attracted great scientists and physicians such as Avicenna, *Abu Sahl 'Isa Ibn Yahya al-Masihi*, to* Chorasmia*. 

Avicenna (980-1037), the famous scholar and physician, conducted scientific research in Ma'mun’s court for 13 years. During this period, he spent his time in teaching, writing, and curing patients. He wrote many books and treatises during his presence in Chorasmia. Two of these treatises in medicine are “Angiology; A treatise on the pulse” in Persian and “*Asbab al-Hozn*” in Arabic, which is about depression (18).

*Abu Sahl 'Isa Ibn Yahya al-Masihi al-Jurjani*, scholar, physician, and mentor of Avicenna, was from Gorganch. Some of his publications on medicine are *al-mā'a fi-l-sanā'a al-tabi'iyyah* (medical encyclopedia in 100 chapters), *al-Teb al-Kolli *(a book about general treatment in 39 chapters), *Ezhar Hekmat allah fe khalgh al-ensan* (a book on human physiology and the purpose of its creation) and *Resaleh* *fe al-jderi va tadbireh* (a treatise about smallpox and its treatment). His other medical researches include *Resaleh fe Amr al-Vaba va al-Ahteraz Anho va Eslahat Eza Vagha* (a treatise about cholera and its prevention), *Resaleh* *fe Tahghegh Soe al-Mezaj Ma Howa va Kom Asnafe* (a treatise on different types of gastric disease), and a book under the title of “Principles of the pulse” (20).

Most of these medical treatises were dedicated to *Abu Abbas Ma'mun II*. Abu Sahl died in 1010 A.D. at the age of 40 when he and his student, Avicenna, were caught in a sand storm while escaping from Mahmud of Ghazni’ agents (21).

Al-Biruni (974-1048) was an astronomer, mathematician, historian, researcher, and a famous botanist from *Birun,* a village near Kath in Chorasmia. He wrote *Kitab al-Saydana Fi'l-Tibb,* which consists of a list of herbal medicine in alphabetic order, in the field of pharmacology in Arabic (21).

*Abolhasan Ahmad Ibn Muhammad* *Soheyli* *Khwarazmi* (1027-?) was a skillful physician and scientist and was appointed as a vizier in Chorasmia for a while. Later, he went to Baghdad, and then, to *Samarra*. Among his medical works are two books under the titles of *Tadrok al-khata fe Tadbir al-Abdan* and *al-Rozat al- *Soheyliat (20).

*Abolkheir* *Khamar* *Khwarazmi* (943-1049 A.D.), physician and famous philosopher, was from Chorasmia. He showed innovation both in theoretical and practical medicine. Among his works are *“al-Hawamel”*, a book on pregnancy, a treatise which was used for testing physicians; and *“Ketab fe* *khalgh al-ensan va Tarkib Aazayeh”* consisting of four treatises. He wrote “*Ketab al-Tadbir* *al-Mashayekh*” which is an adaptation of the work of *Hunayn Ibn Ishaq *(809-873 A.D.) with the same title. Khwarazmi gathered the viewpoints of Galen (Greek physician, surgeon, and philosopher) and Rufus (Greek philosopher, physician, and botanist of Ephesus) in this book. He has a treatise about epilepsy and a book on nutrition (*Kitab al-agdiya*) (20).

Among other physicians from Gorganch who *Professor Fuat Sezgin* talks about is *Abu-Saeid Jorjani* who was a 10^th^ century scholar. He wrote a book on botany which Al-Biruni referred to in his work. Sezgin also mentions *Abu al-Futuh Jorjani*, a physician who lived in the second half of the 10^th^ century, and some other writers have also mentioned his book on medicine (20).

The important point of this period is the fact that in the golden era of the Ale Ma'mun dynasty, many scholars and physicians were attracted to the small and safe geographical zone of Chorasmia (18).

**Third era**

The third era of development of Chorasmia started again a hundred years later with the governorship of *Qutb al-Din Muhammad Anushtegin* (ruling 1097-1128). After him, *Ala al-Din Abul-Muzaffar Atsiz* (ruling 1120-1156), *Taj al-Din Abul-Fath Il-Arslan Ibn Qizil Arslan Atsiz* (ruling 1156-1172), *Jalal al-Din Mahmud Sultan Shah Ibn Il-Arslan* (ruling in 1172), *Ala al-Din Tekish ibn Il-Arslan* (ruling 1172-1200), *Ala al-Din Muhammad ibn Ala al-Din Tekish* (ruling 1200-1220), and *Jalal al-Din Mingburnu ibn Ala al-Din Muhammad* (1220-1231), respectively, governed Chorasmia until the attack of the Mongols. These rulers were under the influence of the Seljuq dynasty for a period of time. However, they moved toward independence after Atsiz, and in addition to Chorasmia, they dominated some parts of Transoxiana,* Khorasan*, *Mazandaran*, *Sistan*, *Kerman*,* Fars,* and *Jibal*. Since the rulers of this dynasty, after Atsiz, were supporters of science, they could gather great scholars and physicians in their imperial court (22).

*Mahmud Ibn Umar* *Jeghmini* *Khwarizmi* (?-1221 A.D.) was a famous physician in *Jeghmin*, which was located in Chorasmia. This scholar and astronomer wrote a book under the title of *“Sharh-e-Qanunche*” which is an abridgment of the *Canon of Medicine* written by Avicenna (23).

*Zayn al-Din Sayyed Isma‘il Gorgani* (1042-1137 A.D.) was born is Gorganch (2,24). Some of his works are *“Khafi Alayee”*, *“al-Tib al-Muluki”*, *“Zubdah al-Tib”*, *“Zakhireh Kharazmshahi”, “Yadegar Dar Teb”,* and *“al-Aqraz al-Tebbieh va al-Mabahis al-Alaieh”*. He presented his works to *Qutb Ad-Din Muhammad* and his son, Atsiz (25). 

Due to writing great medical works, Jorjani became a high rank scientist. First, he was promoted as a governmental senior physician, and then, he was appointed as the physician of *Qutb Ad-Din Tekish*

and Atsiz. He was the manager of the teaching hospital in Chorasmia (2, 25). At the age of 90, Jorjani moved to *Marv* in Khorasan, the capital of Seljuqs, perhaps to improve his scientific work and make use of the large library of the capital. He joined the imperial court of Seljuq ruler, *Sultan Sanjar,* and finally, died in Marv at the age of 95 (2, 24, 26). 

Other physicians of Chorasmia Medical School were *Abu Bakr Abolkheir Ibn Abdur Rahman Jorjani*, *Shahabodin* *Abusaeid* *Khivaghi Khwarazmi,* and *Jamal Ad-Din Qarshi* (1114-1201 A.D.) (27, 28).

After the attack of the Mongols, many of the scholars and physicians were killed or they had to immigrate to Egypt, India, Anatolia, or Fars. This terrible event caused the decline of culture and science in North-East Iran and Chorasmia (29). The last spark of Gorganch was *Sayyed Sharaf al-Din Jorjani* (1340-1413 A.D.) who was respected in the courts of *Shah Shuja Muzaffarid* (ruling 1358-1384 A.D.) and *Timur Beg Gurkhani *(ruling 1370-1405 A.D.). He was the manager of *Dar al-Shafa Mozzafari* Hospital of Shiraz in Southern Iran (30). 

## Conclusion

The presence of great Iranian scholars and physician in Chorasmia (at the time of Ale Iraq, Ale Ma'mun, and the Khwarazmian dynasty) indicates the scientific importance of this geographical zone. This significant presence, no doubt, is a sign of the appealing characteristics of Chorasmia Medical School. The influence of this school can be noticed many years after that era. The scholars of this area were fortunate, since Ale Iraq and Ale Ma'mun governments and the Khwarazmian dynasty all loved and supported culture and science. With their widespread support of research activities, they created a golden era in all scientific fields, especially in medicine.
